# Natural recovery and gaming disorder: a case series

**DOI:** 10.1098/rsos.262017

**Published:** 2026-10-21

**Authors:** Yaewon Jin, Matúš Adamkovič, Marcel Martončik, Veli-Matti Karhulahti

**Affiliations:** ^1^ Department of Content Convergence, College of Science and Industry Convergence, Ewha Womans University, Seoul, Republic of Korea; ^2^ Faculty of Humanities and Social Sciences, University of Jyväskylä, Jyväskylä, Central Finland, Finland; ^3^ Institute of Social Sciences of the Centre of Social and Psychological Sciences, Slovak Academy of Sciences, Bratislava, Slovakia; ^4^ Faculty of Education, Charles University, Prague, Czech Republic

**Keywords:** addiction, longitudinal, qualitative, registered report, technology

## Abstract

Conceptualizations of recovery in gaming disorder (GD) remain relatively underdeveloped. While remission without formal treatment appears common in longitudinal data, the mechanisms through which it occurs have received little systematic attention. In contrast, natural recovery—the resolution of addictive problems without formal treatment—is well documented in substance and gambling research, where it has prompted broader reconceptualizations of how and where recovery unfolds. Using longitudinal qualitative data from Finland, Slovakia and South Korea, this case series examines how individuals who had previously experienced self-identified gaming-related problems reached recovery without successful intervention explicitly targeting their gaming behaviour. Six recurring recovery pathways were identified: axiological shift, social enforcement, gradual regulation, reflexive transformation, healing comorbidity and environmental transformation. Rather than centring on abstinence or direct symptom elimination, recovery emerged through reconfigurations of life structure, social roles and personal value hierarchies. In most cases, gaming did not disappear but was repositioned within a broader ecology of responsibilities and relationships. These findings suggest that natural recovery in GD may be better understood as a contextual and relational reconfiguration rather than solely as symptom reduction. The pathways identified here provide a conceptual stepping stone towards more comprehensive frameworks for understanding the recovery process in GD.

## 1. Introduction

Mental disorders, like many other scientific constructs, are not natural phenomena that exist in the world but socially agreed pragmatic constructs with blurry boundaries (see [1]). A recently recognized mental disorder owing to addictive behaviours, gaming disorder (GD), is one such pragmatic construct, which describes a psychiatric phenomenon for diagnostic, measurement and other purposes [2]. Its inclusion in the *International Classification of Diseases* (11th ed.) has provided formal language for health problems related to video game play; meanwhile, its socially agreed diagnostics, aetiology and symptomatology remain blurred by nature [3]. One mostly unexplored domain in this regard is recovery: in a case where related problems meet a clinical threshold of distress or lead to treatment-seeking, how do such problems resolve? This study offers empirical answers to that question through a series of in-depth case studies.

A central element for understanding recovery in the present context is the fluctuating course of various addictions. For instance, with reference to substance use, Compton *et al.* [4] review the literature and conclude how the ‘trajectory of drug use disorders is not linear but has multiple reciprocal pathways over time between non-use, use, problematic use, and addiction’ [4, p. 381]. For this reason, it can be difficult to assess whether a person's use, non-use or related problems reflect an ephemeral state that is soon about to change or a more stable situation of a life course. Consistent with the above, the diagnostic manuals also acknowledge levels of remission, which reflect the degree to which related problems have been resolved rather than their binary presence or absence. When we refer to natural recovery in the coming pages, all these instances come with a caveat that we cannot know how long the identified state lasts and when it begins to change, if it ever does.

In addiction research, disease models and chronic vulnerabilities may allow remission to be perceived as a stable state, too (see [5]). From such a perspective, a person might never be fully ‘cured’, but there can only be a state of no symptoms. Empirically, this also relates to a so-called general addiction liability, according to which some people tend to be chronically vulnerable to addiction (e.g. [6]). A person may identify themselves as a permanent ‘addict’ (cf. [7,8]) and, as such, be unable to ever engage in gaming, gambling or substances moderately. Even though the person has no related problems, they have a condition that limits their opportunities. In this context, the notion of recovery has a very specific, perpetual meaning.

An alternative approach to addiction perceives its manifestations through choice (e.g. [9]). Accordingly, people do not simply ‘get addicted’ but rather choose to engage in the said addictive behaviour because they experience it—consciously or not—as the best available option they have in life at the moment. In Heyman's [10] words, ‘quitting can only occur if there is a change in conditions that markedly reduces the value of the drug relative to the nondrug alternative’ (p. 130). Here recovery manifests as a change in one's choice-making when other options become possible or more attractive.

The degree to which GD fits in the above frameworks of addiction research continues to be discussed [11]. In this study, we choose not to engage with those conversations directly; rather, we perceive GD as a pragmatic construct that reflects a blurry range of gaming-related health problems. This is consistent with the frequently reported challenges of identifying GD in practice, as genuine efforts of treatment-seeking do not entail meeting relevant ICD-11 or DSM-5 criteria [12,13]. While we acknowledge that such ‘concept creep’ sets limits to our findings [14], we do not believe that the diagnostic construct is currently clear enough to enable ‘true gaming disorder’ as a robust object of study. To combat these construct issues, we aim to describe the gaming-related problems of each case in such depth that current and future readers can situate them in the discourses and diagnostics of their time (see §6.1 for more details).

In what follows, we approach recovery empirically with case study evidence from individuals who had sought treatment for self-identified gaming problems and recovered over time without obtaining formal or sufficient gaming-specific treatment for those problems. We review six cases in detail and outline a distinct natural recovery path for each, which, in turn, are reflected against previous research frameworks. Ultimately, our efforts identify and interpret a set of natural recovery pathways, which can be used as a stepping stone for more comprehensive conceptualization, taxonomization and clinical practice relevant to present and future GD constructs.

**Programmatic registered report**

The present Stage 2 report constitutes a programmatic component of the longitudinal, multi-site Stage 1 registered report *Reconstructing Gaming Disorder: A Taxonomy by Registered Report* [15]. The parent study aims to build a descriptive taxonomy of intensive gaming across three interconnected levels: health, design interaction and the phenomenology of play. Using longitudinal qualitative data from Finland, Slovakia and South Korea, the Stage 1 study continues to chart diverse life scenarios in which gaming plays a significant role. The two research questions of the Stage 1 report are as follows:

RQa: Is it possible to distinguish passionate from pathological gaming by the meanings and values that players attach to video game play?

RQb: What are the design structures of video games, which are played intensively and/or with gaming-related health problems?

This Stage 1 research proposal includes a plan to publish additional case studies/series (pp. 13–14). Owing to the large number of participants and data generated over several years, further details of the planned case studies/series were limited at Stage 1. The plan was to publish at least one study/series per participant group—adolescents, active players of esports games and treatment-seekers, that is at least three in total—selecting relevant cases for closer reporting and using all data types and countries. We planned to make use of an adapted ‘analytic direction’ approach [16] in case selection; namely, to maintain a team-internal list of potential directions that surface over the years, and when enough evidence accumulates for a certain direction to be scientifically meaningful, this was planned to be chosen to guide a programmatic component. Natural recovery turned out to be one such direction. CARE guidelines [17] were planned to be followed in reporting, and this applies to the present case series as well.

Within this broader programme, the present case series represents one of the planned studies that focus on the treatment-seeker group (with one participant from another group selected too owing to a relevant recovery experience in the past). This direction does not fully correspond with the comparative design described in the section ‘Programmatic component 1’ because of a turn in our analytic direction. Nonetheless, most of the planned case study components remain, as detailed below.

The present study is relevant to RQa by examining a trajectory that emerged from the longitudinal data: adult participants who self-reported having experienced gaming-related problems and later described a shift towards a non-problematic relationship with gaming. By documenting how participants themselves articulated the boundary between problematic and non-problematic gaming, and how gaming came to be repositioned within their lives, the analysis provides empirical grounding for the Stage 1 concern with meanings, values and lived experience. We consider this direction to yield the most valuable findings for the field.

The present case series further contributes to the ongoing development of the Stage 1 taxonomy by detailing one emergent trajectory on the phenomenology-of-play level. It also offers future points of connection with the health and design-interaction levels of the taxonomy, particularly by showing how changes in meanings and values are entangled with life functioning, responsibilities, relationships and play routines. Reporting this trajectory at the present stage is useful because recovery from gaming-related problems remains conceptually underdeveloped, while the available longitudinal data already captured cases in which participants described meaningful movement from problematic to non-problematic gaming. The aim is not to finalize the broader Stage 1 taxonomy before the completion of the study but to document an emergent trajectory that can inform its ongoing development.

As a Stage 2 registered report, this study draws methodologically on the longitudinal design, recruitment procedures, data collection and provision for programmatic components outlined at Stage 1. However, the specific focus on natural recovery, the selection of cases and the pathway structures were generated inductively from the data. Further details are provided in ‘Data and methods’.

## 2. Natural recovery in addiction

Natural recovery, as a concept, is potentially applicable to almost all mental disorders and has a wide-ranging literature, for instance, in the study of depression (e.g. [18]). Owing to various differences between psychiatric phenomena, such as those concerning mood, neurodevelopmental and psychotic disorders, below we review relevant natural recovery literature with an explicit focus on addictive and substance-related disorders. We consider this specific focus justified because addictive behaviours and substances remain the diagnostic category through which GD is currently diagnosed.

In addictology, natural recovery generally refers to the ‘remission or resolution of addictive problems without formal/specialized treatment or mutual-help participation, often through self-initiated changes in use and life circumstances’ [19] (see also [20–23]). Closely related terms, including spontaneous remission, unaided recovery, self-change and untreated remission, have been used across disciplines to describe similar phenomena [24,25], reflecting differences in operationalization rather than substantive conceptual disagreement.

The roots of this idea can be traced to mid-twentieth-century research, most notably Winick's [26] analysis of heroin users, which introduced the notion of ‘maturing out’ to describe substance use that sometimes declined as individuals’ life circumstances changed. This early recognition of addiction as, at least in some cases, a self-limiting or developmentally contingent process laid the groundwork for subsequent research. For decades, addiction remained predominantly conceptualized within a chronic and progressive disease framework, and recovery without formal treatment was often viewed with scepticism. As Chiauzzi & Liljegren [27] noted, ‘an ability to cease addictive behaviors on one's own suggests that the individual was not addicted in the first place’, whereas continued inability to stop was taken as confirmation that addiction was present (p. 306). Such logic is evidently tautological, nonetheless, and was accordingly challenged in various scientific fields.

Over time, accumulating evidence across substances led to growing institutional recognition of recovery outside formal treatment, reflected in reports by the Institute of Medicine (1990) and discussions within the American Psychiatric Association (1994) [25]. By the late 1990s, the concept had extended to behavioural addictions, particularly pathological gambling. The National Research Council [28] acknowledged that ‘recovery from pathological gambling can take place without formal treatment’, while noting that this possibility had received limited prior attention (p. 193). Subsequent community-based longitudinal studies further demonstrated that gambling problems often follow a transient or fluctuating course [29]. What was once considered a ‘taboo’ notion [27] is increasingly recognized, particularly in alcohol and gambling research, as a common pathway of problem resolution [20,30].

The empirical recognition of natural recovery has prompted two related shifts in addiction research. First, recovery is increasingly understood as taking diverse and multidimensional forms [31,32]. In alcohol and policy discourse, recovery has moved beyond being equated solely with sobriety and is now often defined as ‘a process of change through which individuals improve their health and wellness, live a self-directed life, and strive to reach their full potential’ [33, p. 3]. In gambling research, definitions likewise vary, encompassing abstinence, absence of diagnostic criteria, reductions in severity and improvements in functioning, with substantial heterogeneity in how recovery is operationalized across studies [34,35].

There has also been a growing interest in the mechanisms of recovery that operate outside formal treatment systems. A range of processes have been identified in this regard, including maturing out, adaptive or self-regulated use, cognitive reappraisal, engagement in alternative activities and new social roles, lifestyle and environmental transitions, and the mobilization of social and material resources [20,23,24,29,36–41]. Although diverse, these mechanisms converge in relocating recovery from discrete clinical intervention to broader transformations in life structure, identity and social context. Seen in this light, natural recovery is better understood not as a single event but as a temporally extended process unfolding over time. Qualitative and longitudinal accounts describe recovery as unfolding through repeated attempts, lapses, turning points and gradual stabilization of new behavioural patterns [38]. Explanations frequently emphasize environmental and lifestyle changes, suggesting that shifts in daily routines, social roles and broader life circumstances can alter the opportunity structures that sustain addictive behaviour [23].

Given this emphasis on resources and context, the concept of recovery capital becomes particularly salient. Rather than framing recovery solely as the resolution of individual pathology, this approach reconceptualizes it as a resource-mediated social process [20]. Influenced by sociological notions of capital, Granfield & Cloud [37] defined recovery capital as ‘the sum of one's total resources that can be brought to bear in an effort to overcome alcohol and drug dependency’ (p. 179). The concept has since been elaborated to encompass multiple domains of personal, social and community resources, including physical health, financial stability, safe housing, coping capacities and recovery-supportive relationships [42]. Framed ecologically, recovery unfolds through the dynamic interaction of individual agency and enabling environments rather than within the person alone [43]. As a concept, recovery capital remains closely related to the notions of resilience and protective factors, which have also been addressed in GD research (e.g. [44]).

In sum, natural recovery is not merely a descriptive phenomenon but a conceptual development that has contributed to rethinking how recovery is defined and where its mechanisms are located across substance and behavioural addictions. In the case of gaming, longitudinal research increasingly indicates that a notable proportion of individuals no longer meet diagnostic thresholds over time, even in the absence of professional intervention, with remission rates in some studies approaching one-third to one-half at follow-up (e.g. [45–47]). Complementing these findings, a 5-year Korean panel study tracking approximately 1800 participants from childhood to adulthood reported that none of those classified in the high-risk gaming group at the start remained continuously in that category across the full follow-up period [48]. These findings indicate that GD remission without formal treatment is not uncommon.

Yet, compared to the more developed recovery discourse in alcohol and gambling research, conceptual frameworks capable of accounting for non-treatment-based recovery remain relatively limited within GD scholarship. Indeed, a recent scoping review of GD recovery found that out of 47 studies, only 5 explicitly addressed ‘recovery’ as a concept; all others simply measured decreases in gaming symptoms or related psychopathology [49]. This pattern suggests that recovery is largely inferred from symptom reduction or treatment outcomes rather than conceptualized more broadly as a contextual and developmental process [50].

This gap is particularly notable given the distinctive features of gaming. Unlike substance use, gaming is deeply embedded in everyday digital infrastructures and often serves diverse psychosocial functions, including social connection, achievement, identity work and emotional regulation [51–55]. These characteristics suggest that GD recovery may involve not simply cessation but the renegotiation of gaming's place within relational networks and daily routines. Whether natural recovery from gaming-related health problems mirrors mechanisms identified in substance use disorders, or instead follows distinct relational and contextual configurations, remains insufficiently examined. Addressing this question is critical not only for theoretical coherence (as per the position of GD in addiction research) but also for informing public health responses that avoid over-pathologizing transient or developmentally contingent patterns of gaming. The present study adopts a cross-cultural qualitative case-series design to examine how natural recovery unfolds through relational and contextual reconfigurations in lived trajectories.

## 3. Data and methods

The cases of this study are selected from an ongoing 5-year longitudinal research project, which received ethics approval in Finland (University of Jyväskylä, ref. no. 1054/13.00.04.00/2022), Slovakia (University of Presov, approval issued 14 September 2022) and South Korea (Yonsei University, approval no. 7001988-202303-HR-1829-02), respectively. All participants provided written informed consent prior to participation. Positionality statements for all team members are publicly available at https://ore.jyu.fi/english/our-team. The data generation and analysis plans were reviewed as a Stage 1 registered report [15]; full descriptions of the study design, participant groups, recruitment procedures and data generation instruments are provided in appendix 1 of the supplementary material.

The present study was initiated after diverse trajectories of problem resolution had manifested in the longitudinal data, offering a unique opportunity to examine recovery processes that remain understudied. We recognized that little to no research had been carried out on these processes, and our in-depth longitudinal design was well positioned to capture them. The study was conducted in accordance with the Stage 1 approved protocol.

The overall data relevant for the present study include adults from two groups: treatment-seekers (defined as those who had sought clinical help for gaming-related problems either as self-initiated or persuaded by someone) and competitive players (defined as those who were playing esports games more than 30 h per week without any current gaming-related health problems).^1^ Gradual participant recruitment started in 2022 in Finland, Slovakia and South Korea, and after having met the recruitment goals in 2024 (table 1), the longitudinal process of following all participants for 3 years remains ongoing until 2027 (total adult *n* = 78).

**Table 1.** Distribution of participants by country and recruitment group.

	Finland	Slovakia	South Korea
treatment-seekers	*n* = 18	*n* = 15	*n* = 19
competitive players	*n* = 5	*n* = 14	*n* = 7

*Note:* Following the registered research plan, the sampling frame for treatment-seekers was extended to include neighbouring Nordic (for Finland) and Central European (for Slovakia) countries after completing local recruitment (*n*_Finland_ = 16; *n*_Slovakia_ = 5).

We screened all adult participants (*n* = 78) for eligibility for the present case series by reviewing phenomenological interview transcripts from the first two annual cycles. The third annual cycle remained ongoing at the time of analysis (see appendix 1 for the full data generation protocol). Inclusion required the following criteria: (i) a previously reported period of self-identified gaming-related problems, accompanied by perceived difficulty regulating gaming and/or disruption in academic, occupational or relational functioning; and (ii) subsequent resolution of these problems without receiving formal or sufficient treatment specifically targeting gaming behaviour. We use ‘formal or sufficient gaming-specific treatment’ to refer to a structured professional intervention primarily targeting gaming behaviour, including psychotherapy, psychiatric treatment, counselling or a treatment programme in which gaming was the explicit focus. Resolution was defined as the participant's self-reported perception that gaming was no longer experienced as problematic or interfering with valued responsibilities or daily functioning (but see our caveats on the natural course both above and below).

Of the 78 adult participants, 22 met both criteria (figure 1). The remaining 56 participants were not eligible because they did not report a prior self-identified gaming-related problem, did not report subsequent resolution or had received structured gaming-specific treatment. It may seem paradoxical that treatment-seekers—defined as those who sought help for their gaming—are included in a study of untreated recovery. However, treatment-seeking and receiving structured gaming-specific treatment are not equivalent. Within the treatment-seeker group, participants varied in the extent to which they had received help: some were seeking treatment unsuccessfully, whereas others had sought help but could not access appropriate services; others had received counselling or clinical support focused on comorbid conditions such as depression or anxiety rather than gaming behaviour itself. The discovery of such great variety in what treatment-seeking meant in practice was, in fact, one of the key factors that contributed to our analytic direction moving towards (natural) recovery.

**Figure 1.**
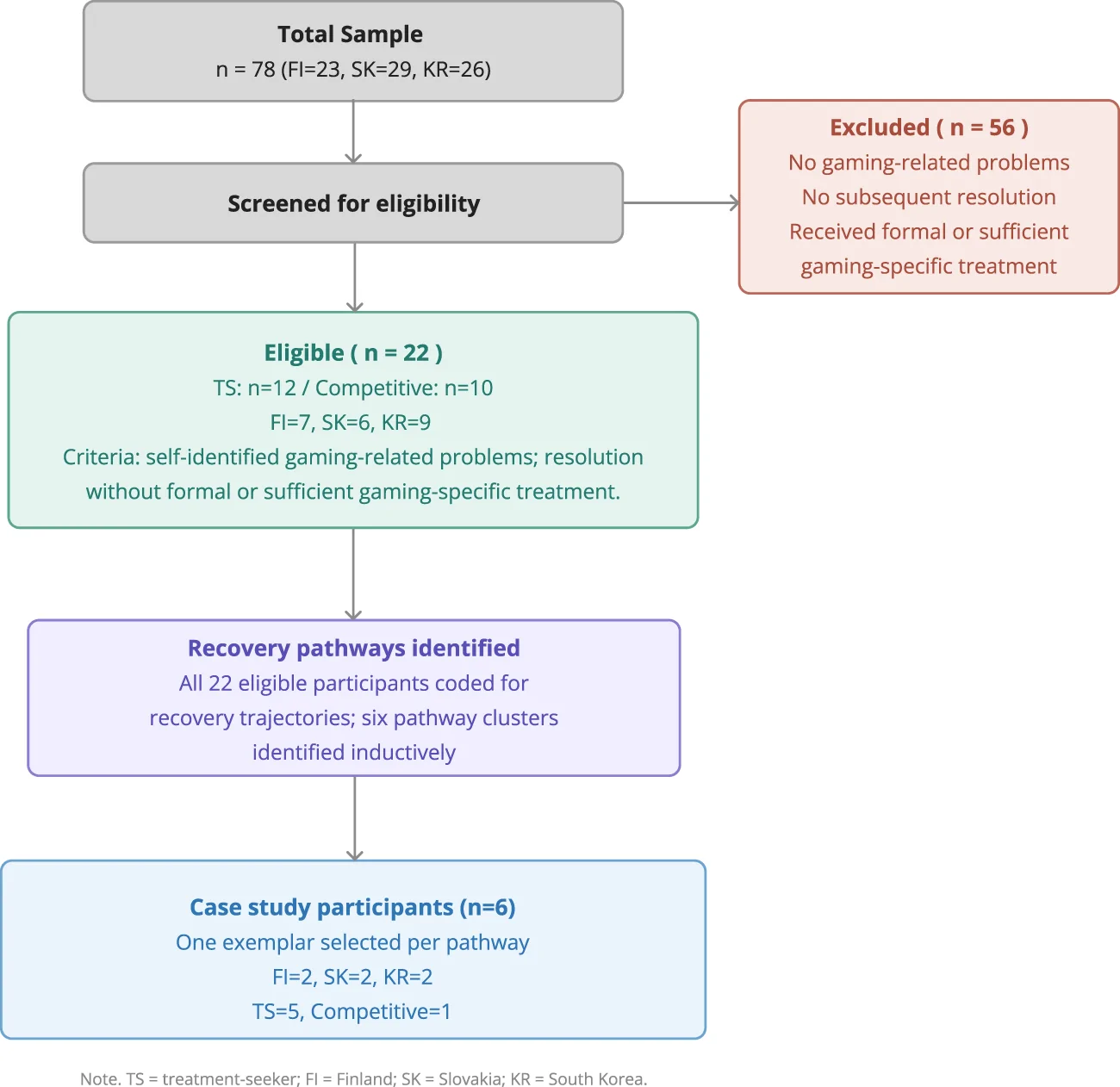
Participant screening and case selection.

The above criteria are consistent with established definitions of ‘untreated’ or ‘unassisted’ recovery in the natural recovery literature where recovery is considered untreated in the absence of problem-specific treatment or mutual-help participation, regardless of broader healthcare utilization [22,24,29]. For instance, participants who had received support targeting comorbid conditions (e.g. depression and ADHD) were not excluded.

For all 22 eligible cases, we coded their pathway from problems to recovery. Initial coding was conducted within country teams in the original language. Korean cases were coded first and generated a provisional set of sensitizing concepts. Slovak cases were then coded with reference to, but not constrained by, these provisional concepts, while Finnish cases were coded independently. Across all sites, country teams remained free to revise, reject or add codes based on the idiographic features of each case. Codes were then compared across contexts through iterative discussions among the authors, and recurring patterns were identified (supplementary material, appendix 2).

This process resulted in six recurring pathway patterns, which thus represent distinct recovery pathways that have some case-level variation in our data. We present each pathway via a selected participant (table 2). Exemplar participants were chosen through team consensus applying the following criteria: (i) the case clearly illustrated the identified pathway; (ii) the participant's data were rich across interview rounds; and (iii) the pathway was expressed as a primary trajectory, rather than deeply intertwined with other pathways.

**Table 2.** Identified natural recovery pathways.

**pathway**	**description**	**case study**
axiological shift	gaming falls down in one's personal value hierarchy	Beáta, competitive player, Slovakia
social enforcement	compliance with people of influence	Kangmin, treatment-seeker,^a^ South Korea
gradual regulation	self-control is obtained over time	Gabriella, treatment-seeker, Finland
reflexive transformation	a moment or event triggers life change	Lauri, treatment-seeker, Finland
healing comorbidity	gaming changes along with a cured illness	Gunho, treatment-seeker, South Korea
environmental transformation	gaming changes with the environment	Pavol, treatment-seeker, Slovakia

^a^ Kangmin was recruited into the treatment-seeking group as he self-reported having sought gaming-related treatment in the screening process. However, interview data revealed a more nuanced picture: while he had sought professional help, this was not primarily gaming-related, and he had considered professional help for gaming but had not actively sought it. We therefore retain his original recruitment-based classification while reporting this discrepancy transparently. For the purposes of the present case series, Kangmin meets the analytic inclusion criteria because his gaming-related problems were resolved without gaming-specific intervention.

Two participants were selected from each of the three countries (Finland, Slovakia and South Korea) to support cross-cultural reflection; authors of each respective country analysed each case in the original language to improve interpretive depth. Although all six cases were based on the first two annual cycles of data collection, the quantity and depth of available material varied depending on how extensively participants shared their experiences across interviews and diary materials. In this case series, each participant therefore serves as a single instance of a specific natural recovery pathway, rather than as a statistically representative case.

Importantly, the identified pathways are not mutually exclusive. A person can find a recovery route that consists of at least one but up to six pathways combined. As the cases demonstrate, indeed, for some participants, recovery involves several mutually interacting pathways that collectively led to completed or partial remission. We discuss the relevant synergies further in the discussion.

In the case presentations that follow, we have used all the generated data from each participant to produce informative examples. Phenomenological interviews served as the primary data source for idiographic case construction, following IPA principles ([56]; see appendix 1). Clinical interview transcripts, gaming diary entries, panel data and psychometric reporting were reviewed across all available time points and used to contextualize and enrich the case descriptions.

The data are currently being archived to the Finnish Social Data Archive and will be available for scientific reuse in pseudonymous form.

## 4. Results

### 4.1 Six natural recovery pathways

#### 4.1.1. Axiological shift: ‘Beáta’

Beáta is a woman in her early 20s from a small Slovak town. Her parents separated, and her father died thereafter when she was still a teenager. This familial instability was coupled with social isolation. Beáta has been diagnosed with OCD and self-reports occasional bursts of aggression and meltdowns. From early childhood onwards, she spent a substantial amount of time playing video games and gradually structured her life around competitive gaming. She recalls periods when gaming became intertwined with her OCD: when she was unable to complete quests or achieve in-game goals, she experienced meltdowns and seizure-like episodes. Over time, gaming became the primary domain in which she felt competence, continuity and positive affect. Up until recently, gaming also served as a means of social connection with her cousin and a group of friends.

At the time of the first interview, Beáta was enrolled in a STEM programme, which she described as both difficult and dissatisfying. Rather, she expressed a strong interest in activities such as design, drawing and character modelling, with the aspiration to create her own storyworld. After 1 year, she decided to leave the programme and subsequently managed to get enrolled in an art and design programme. This transition represented a major turning point. Beáta framed this change explicitly as a priority conflict:

The ranking [in competitive gaming] stopped being so important because I had to study for exams. It just didn't interest me that much anymore … the school I am in now actually interests me … it feels easier, I enjoy it more, and I understand it better.

As a new interest entered her priorities, gaming's position within her value hierarchy changed. Academic responsibilities—now intertwined with creative self-realization and game-related activities such as digital design, drawing and 3D modelling—became internally meaningful. She reduced her gaming time by half, now playing around 10–20 h per week. She became increasingly future-oriented, focusing on completing her degree. Gaming had become a source of relaxation rather than a central organizing activity.

Maybe I play for shorter periods, or I spend more time drawing and modelling 3D things; and in terms of my style of gaming, it is more casual and not try-hard. I try less to do it perfectly and try more to have fun.

Her gaming did not lose its entire value but merely took less time to secure space for more intrinsically rewarding and creative activities. She described this shift as a reordering of priorities: ‘To finish school, that's probably the biggest value for me right now.’ Overall, Beáta's case illustrates a gradual reranking of priorities, in which gaming increasingly conflicted with activities she perceived as more meaningful, future-oriented and identity-relevant. Gaming, however, remained enjoyable.

**Sociocultural context**

*Beáta's axiological shift can be perceived through a Slovak context in which gaming was socially available but only ambivalently legitimized, let alone as a serious educational or career path. In her family, gaming was tolerated as a hobby but subordinated to school, sleep, outdoor activity and ‘normal’ social development. Her trajectory reflected this tension: despite long-standing interests in games, drawing and creative production, she first entered a more conventional university track before transferring into digital games. This transfer placed gaming within a more legitimate educational and vocational framework, where it could be connected to design, 3D modelling and future work. At the same time, she viewed wider Slovak society as largely dismissive of gaming and esports and described multiplayer gaming spaces as male-dominated and often hostile. Over time, gaming was repositioned from privately competitive leisure activity into a culturally respected creative pathway organized around school, design and future work*.

#### 4.1.2. Social enforcement: ‘Kangmin’

Kangmin is a 41-year-old Korean man who lives alone. He identifies his late 20s, during graduate school, as the period when his gaming was most intensive. For 2–3 years, he recalled spending nearly 12 h a day at a PC bang almost every night, playing Sudden Attack with friends. During this period, gaming became a fixed overnight routine and a central organizing feature of his daily life.

He did not find such a pattern problematic at first. This view changed later from a sense that daily life was no longer functioning well. During the final stage of his graduate programme, in particular, long gaming hours distracted his academic progression severely. He experienced physical fatigue, emotional instability and disrupted daily routines. He described this period as a time when he felt he could not continue living this way.

He considered seeking treatment and was aware of it as an option. However, he did not pursue it, despite already having had treatment experiences unrelated to gaming; Kangmin had previously received self-analytic counselling for self-understanding, relational difficulties and general psychological discomfort. He later took medication for work-related stress and sleep difficulties. Neither was sought for gaming-related problems.

At the time, I did think about it … whether I should get outside help. … Because my major was in that area, I knew where I could go. Addiction-related services, counseling centres, or hospitals were all options I had in mind. … But there was the hassle of having to look for it. I knew the kind of place I should go to, but I still had to find where exactly to go.

He cited the inconvenience of seeking help and his belief that he would not live like this forever. Kangmin's recovery was enacted when a figure of authority explicitly intervened and reasserted normative expectations. His thesis supervisor, a figure of major influence and authority in the Korean cultural context, reached out to him and insisted that he should start writing.

With one semester left before my thesis, my thesis supervisor contacted me and said, ‘Shouldn't you be writing your thesis?’ and I answered, ‘Yes, I should.’ And once there was the thesis, I felt that I did not really have to keep doing this [gaming] anymore.

The intervention reintroduced institutional expectations that Kangmin could no longer ignore, making the continuation of his gaming no longer defensible. His gaming had also been embedded in a peer routine with older male friends and classmates, but this routine became less viable as those around him also ‘had to start living their own lives and writing their theses’. In this sense, the supervisor's intervention coincided with a broader relational transition in which intensive gaming no longer fit the shared temporal structure of his peer group.

After this period, Kangmin did not completely stop gaming but rather restructured his relationship with it. Over the following 10 years, he did not return to long-hour gaming patterns. Today, he continues to limit gaming time to 2 h per day and no longer feels that gaming interferes with daily life or responsibilities.

**Sociocultural context**

*Kangmin's recovery through social enforcement was embedded in South Korean academic culture, where a supervisor's authority carries strong normative weight and academic responsibilities are deeply internalized. His problematic gaming had developed within PC bang culture where intensive gaming can function as a social routine among young Korean men. This also meant that gaming became less sustainable once the peer group moved on. In this context, social enforcement operated through the combined force of his relationship with his supervisor, his PC bang-based peer relations and broader social expectations around academic progress and adult responsibility, making continued intensive gaming difficult to sustain*.

#### 4.1.3. Gradual regulation: ‘Gabriella’

Gabriella is a woman in her 60s. She recently retired from work owing to health reasons, as problems with her back significantly restrict mobility and make many tasks painful. After retirement, she suddenly had plenty of time available during the days. Gabriella enjoys spending time on housework, and she reads several hours per day (both fiction and news), and her social life involves spending time with her children and relatives. In her own words, she has a tendency to ‘overthink’ when many things—especially during stressful times when still working—get stuck in her head. Gabriella had never played video games in her life, but one day a friend recommended her to start playing online Mahjong, which could help her to relax. Gabriella followed the advice and started playing every morning.

At first, gaming served the intended purpose well. Gabriella found that it added meaning to her life, as she played one selected Mahjong every morning. Although this was a personal, single-player experience, the browser application also presented her with rankings where she could see her own results against others. For reasons that Gabriella cannot explain, she developed a need to obtain the first place in the ranking every morning: she played as many rounds as needed to get to the first place and did not play afterwards before the next day. Over time, however, she found out about other Mahjong applications too and gradually added them to her morning routine. At one point, she played no less than eight different Mahjongs, and—for each—she had to achieve the top score every morning. On some days, Gabriella could finish playing it in just 1 or 2 h, but on other days, it could take as long as 8 h or more. In the latter case, she found herself with guilt and regret, as it felt like the entire day was gone for nothing.

It annoys me, but I still do it, and then I'm really angry with myself, like, ‘Yeah, okay, you could have baked’, or ‘you could have’ … So I leave those kinds of things—because I like baking, cooking—but I'm leaving those kinds of things, especially now that I'm alone. So everything else gives way to gaming. And then I'm angry, like, ‘Okay, it went like this again.’

The daily rankings had lost their meaning, and she no longer had sufficient time for the daily tasks that she personally found meaningful. The above situation continued for a few years, and she considered having developed a gaming problem. Gabriella wanted to reduce her time spent on gaming but did not know how: she was looking for help to resolve the situation, but it was too embarrassing to share with a clinician. When she noticed the call for participation in GD research, she was enthusiastic to share the details of her problem in interviews.

After a year of participation, however, Gabriella expressed having learned to manage her gaming. Gradually, she had dropped one Mahjong after another and was no longer playing but one or two daily. This took her typically just 1 h in the morning, which she felt was a good fit for her daily morning routines.

I don't feel like I should decrease it even more now … I'm quite astonished by the situation. The idea that I'd play 8 games today, like even the idea of it distresses me. I can do with less nowadays. (laughs) I don't-, know-, I don't know how to explain it, but the opposite reaction feels so surprising, like something that has been an obsession to me feels bad now, *seriously*, if someone told me I have to play now … it would feel bad, like, oh, I have to do this. (laughs) I don't know why, why it's like that, but I'm happy that it is, happy that it has happened.

When discussing how Gabriella had learned to regulate her gaming, she said to have actively engaged with the anxiety that gaming had generated for her. She had come to reflect on the negative feelings that the need to make top scores had triggered, and she had thus decided to start giving up these obsessions actively. In the third round of interviews, after 1 more year, she had kept playing her two selected Mahjong games and was happy to have found a balance with her morning routine.

**Sociocultural context**

*Gabriella's case takes place in Finland where the climate causes major seasonal variation. In Gabriella's region, the wintertime is characterized by long periods of darkness and icy weather, which negatively contributes to her mobility. She cannot go out without a great risk of falling during the long winters, and the lack of sunlight further makes staying at home an attractive daily option against the alternatives. Combined with a strong ethos of independence in Finnish culture—people do not typically live with their offspring when they age but rather alone or together with a partner—gaming becomes almost a natural activity that fills time.*

#### 4.1.4. Reflexive transformation: ‘Lauri’

Lauri identifies himself as an introvert and not socially skilled. He enjoyed gaming already in primary school where an MMO provided him with access to a peer group and helped him to connect with friends. After some time, however, the peers quit gaming, and Lauri was left to play the MMO alone. His parents divorced, and he was diagnosed with depression. Lauri could play literally around the clock—a dozen or more hours in a row—and retrospectively he perceives that as depression self-medication. After the MMO, he started playing various single-player strategy games. For Lauri, gaming was not a social but a personal space where he could have full control of the events that he participated in.

Lauri finds starting school, work and other such responsibilities extremely difficult. Especially when at home, it feels much easier to start or continue gaming instead of carrying out the responsibilities, which he nonetheless acknowledges as things that he personally values and would want to do. The same applies to social life too: he would like to have more and deeper friendships, but it is just easier to keep playing the strategy games, which he is familiar with and where things are in control. When Lauri entered a vocational school, he found himself choosing gaming over schoolwork repeatedly, which ultimately led him to fail a class and drop out from the programme entirely. He identified gaming as a problem and sought help from support groups and clinical experts but did not find them resolving the problem.

A year after Lauri had joined this research as a participant, he found a new school which he cares about a lot. The schoolwork was thematically related to the strategy games that he had played. Soon, however, it seemed that history would just repeat itself: Lauri found himself gaming and postponing schoolwork to such an extreme that it was impossible to finish class assignments in time. One specific day, he realized that it was too late to catch up with the class before the deadline. This was a transformative moment: Lauri felt a strong emotional experience, as the memories of his past failures surfaced all at once. He became angry at himself and his incapacity to prioritize the things he values. At that very moment, Lauri quit gaming almost entirely.

VMK: Can you reflect on that a little bit, what do you think caused that change?

Lauri: I think a concrete change happened in the new year. I had procrastinated completing a course for school for too long. That kind of upset me, and I had to start taking school more seriously.

VMK: What made it feel so impactful at that moment?

Lauri: Well, I've dropped out of school before, so I think this brought back the memories, like, is something like that going to happen again … Maybe it took something, like almost failing one course. That was the point that, like, forced me to actually try and start focusing.

He made a decision to focus his energy fully on the responsibilities in school and work, and for the remaining time, he started active exercise and enrolled for new social activities. At the time of writing—yet another year later—Lauri's transformation holds. He enjoys being successful in both school and the related work he does on the side, and when he allows himself a strategy gaming session now and then, it feels relaxing and never exceeds two hours.

**Sociocultural context**

*In contrast to Kangmin (and Gunho below), to whom gaming occurs in parallel with major educational pressures, Lauri's life and studies happen in Finland with less societal pressure. Even though education is valued in Finland, the system is not as competitive as in countries like South Korea, and carrying out one's studies is a matter of independently taking responsibility for one's life. Studies are also supported financially by the Social Insurance Institution of Finland, which provides some income for students, next to other potential sources of funding. This context of freedom and independence did not fit Lauri in the earlier years, as he was unable to create and follow schedules that would meet the expectations of the school. This changed after his reflexive transformation*.

#### 4.1.5. Healing comorbidity: ‘Gunho’

Gunho describes gaming as deeply intertwined with his personal interests, sense of identity and anticipated career path. From a young age, he spent long hours playing video games and continued to do so despite parental attempts to restrict his gaming. Over time, he became aware of his difficulties with self-regulation. Gunho identified his first year of university as the most problematic period in his gaming trajectory. The school transition coincided with his first experience of living in a dormitory, where daily structure and external control were largely absent. His gaming escalated, and he increasingly neglected his academic responsibilities, eventually leading him to secretly take a leave of absence from the university, while lying to his parents that he was studying.

He described this period as feeling ‘completely broken’. For Gunho, gaming became problematic not because of the amount of time spent playing but because it functioned as a means of task avoidance and stress regulation. The problem emerged when he had ‘something to do’ but avoided it and ‘kept postponing it by playing games’. Gaming prevented him from finishing something he had intended to complete.

I was never formally diagnosed with depression. It wasn't that I felt deeply depressed, but when I took psychological assessments, the results seemed to fall somewhere between normal and mild depression. From my perspective, my situation at the time was really bad—my relationships had fallen apart, I had intense self-loathing, and everything felt unstable.

Although Gunho was not formally diagnosed with depression, he described a period of significant emotional distress that coincided with his most intensive gaming period. Gunho started to hate himself for continuously gaming and decided to participate in psychological counselling offered by the school. Gunho initially sought counselling because of gaming-related distress, but the counselling he received was general psychological counselling rather than structured gaming-specific treatment. Through year-long counselling, he gained a better understanding of his personality and developmental background, but counselling was expensive and did not help him reduce gaming.

The key turning point was the diagnosis and treatment of a comorbid condition, ADHD, which was diagnosed soon after. After starting ADHD medication, he reported improvements in attention and self-regulation:

After taking Concerta, the frequency of avoidant gaming decreased a lot. … And for the first time in my life, I was able to stop gaming for a while, although it was only for about three weeks. … But after about two weeks … I felt that if I suddenly stopped gaming, so much of my life was being negated. … So I thought I should try to enjoy games in a healthy and well-regulated way.

As complete abstinence from gaming disrupted his sense of identity and life continuity, he did not quit entirely but continued in a more regulated manner, with the help of the new medication. At the time of the last interview, Gunho was enrolled in a master's programme and played video games for approximately 10–20 h per week. However, gaming no longer functioned as a strategy for task avoidance nor organized his daily life.

**Sociocultural context**

*In Gunho's case, intensive gaming emerged after a transition out of South Korea's exam-oriented high school culture, where daily life is often restrictive, study-centred and tightly organized around preparation for university entrance. After university entrance, and especially after moving into a dormitory, this externally regulated routine was replaced by sudden autonomy. While this transition is not unique to Korea, the cultural weight attached to academic achievement, reflected in his concealment of a leave of absence from his parents, meant that his difficulties carried not only personal but deeply social significance, positioning gaming as a site of task avoidance, emotional escape and self-blame*.

#### 4.1.6. Environmental transformation: ‘Pavol’

Pavol is a man of approximately 40 years of age, married and pursuing a part-time doctoral degree alongside his employment. He works in the creative arts sector, where he is self-employed. His work requires the use of a computer and talking to clients on the phone, handling various tasks, and is largely conducted from a home office. He typically works on his computer with a video game open in the background, making it easy to switch between work and gaming and back.

Initially, he played during time designated for work—that is during his home office hours—for approximately 30–120 min. In the evenings, he continued ‘often until complete exhaustion’, which would extend into the early morning hours. His primary hobby is listening to and creating music, including performing live. His social contact occurs primarily through music, parties and concerts. These parties are associated with the use of soft drugs and excessive alcohol consumption, with which he had experienced problems. Music is markedly important to him also because of its social dimension—the community surrounding his genre, the parties and concerts. Music represents ‘belonging, that it is something through which I can also connect with people’, and he continues,

At the same time, I have the feeling that [creating music] is something that is simply more socially acceptable in my community and surroundings, that it is simply a socialization tool, whereas gaming is more of an escape, perhaps.

After a year, significant changes occurred. He started collaborating with an internationally known artist.

It is really a meaningful thing for me. So … I really give it priority … because I enjoy it extremely. … The volume of responsibilities has also increased, and that probably naturally keeps me in check in other things as well.

He decided to stop drinking alcohol: ‘I have the feeling that I somehow naturally arrived at it, that I told myself, well, I've had quite enough to drink already, and at the same time, the alcohol really wasn't bringing me any particularly good experiences.’ He explains that it is not a matter of denying himself alcohol but rather ‘that I arrived somewhere and realized, oh, I actually don't need this at all’. He describes this as a liberating feeling: ‘But yeah, I feel like probably the best I've ever felt right now, and it's actually a very, very nice feeling.’

Pavol's decision to stop drinking initially appeared unrelated to gaming. However, it produced a cascade of changes across his everyday life: sleep improved, his relationship with his wife became more stable, a lucrative work opportunity increased economic security, and he and his wife were able to spend more shared leisure time outside the home. Alcohol abstinence became the entry point for a broader reorganization of the everyday conditions in which gaming had previously functioned as escape. As these conditions stabilized, gaming shifted to a more peripheral and deliberate activity.

[Gaming] is also something that I feel I actually have under control now. And basically since I stopped drinking, I feel an overall greater sense of control over all other forms of addiction, right, whether it's gaming or, say, those soft drugs or or some other forms of escape.

Gaming retains the same meaning for him; he describes it as a ‘gamer escape’ or ‘unwinding and releasing the stress and and … focusing on something else for a moment’. During home office hours, he plays less frequently, and when he does, it is a game in which matches are short. Evenings are his time for more extended play, but he states that it is always a rational choice, because he knows that his work is done, and if he plays longer into the night, it is over the weekend, when he can afford to do so.

In a follow-up interview the following year, Pavol reported a difficult life situation—a deterioration of his relationship with his wife and a planned divorce. This again triggered excessive gaming: ‘I played [game title], over and over, a classic “binge session”. At the same time, I was smoking weed.’ He interprets this state through self-reflection as follows: ‘gaming is a symptom of these problems—escape as a form of “defence” is something I do’. Following a period of excessive gaming (‘binge sessions’) into the early morning hours, Pavol decided it would be better for him to sell his gaming computer—which he subsequently did—as he recognized his inability to maintain control over his gaming during such critical life phases. This recurrence suggests that Pavol's gaming was highly sensitive to environmental stability. When the everyday conditions that had made gaming manageable deteriorated, gaming again became an easily available form of escape. By later selling his gaming computer, Pavol tried to alter the environmental conditions that had facilitated binge gaming.

**Sociocultural context**

*Pavol's environmental transformation can be understood within an urban Slovak creative-culture context in which music, clubs, festivals and nightlife were central sources of work, identity and belonging. This milieu made alcohol and soft drug use socially available and partly normalized, while music remained a legitimate form of social connection and professional self-realization. Gaming, by contrast, was meaningful but more private, solitary and framed by Pavol as an escape. His recovery therefore did not involve leaving his sociocultural world, but changing how he participated in it. As alcohol abstinence aligned with his new professional responsibilities, he could remain connected to music and nightlife without relying on intoxication. In this transformed environment, work responsibilities, music, his relationship and shared leisure outside the home became stronger organizing forces, while gaming shifted from an easily available escape into a more deliberate and controllable activity*.

## 5. Cross-case synthesis: overlapping recovery paths

The six recovery pathways identified in this study do not function as mutually exclusive categories, but rather as interacting dimensions. In some trajectories, one pathway appears central, while others remain peripheral; in others, multiple pathways operate in parallel or emerge sequentially over time. Taken together, the six pathways allow for more than 60 possible recovery combinations, where the six contributing dimensions can be more, less or not present. These combinations are not fixed typologies but illustrate how the pathways interact to produce distinct recovery configurations across cases ( table 3). Below, we discuss selected cases to demonstrate how these configurations manifested in practice.

**Table 3.** Overlapping recovery pathways and their relative influence across cases.

recovery path	**Beáta**	**Kangmin**	**Gabriella**	**Lauri**	**Gunho**	**Pavol**
**axiological shift**	strong	some	some	some	strong	strong
**social enforcement**	little	strong	little	little	little	some
**gradual regulation**	some	some	strong	strong	strong	little
**reflexive transformation**	little	little	some	strong	little	some
**healing comorbidity**	some	little	little	little	strong	strong
**environmental transformation**	strong	strong	little	little	strong	strong

*Note:* Influence levels (strong, some and little) reflect the relative functional role of each pathway within the recovery trajectory. ‘Strong’ denotes a central organizing role, ‘some’ indicates a meaningful but secondary contribution, and ‘little’ refers to a peripheral or indirect presence rather than complete absence. These categories are interpretive and do not represent quantified or causal weights.

In the case of Pavol, we have highlighted environmental transformation because his recovery was accompanied by broader changes in everyday conditions, including improved sleep, a period of more stable relationship with his wife, increased financial stability after a lucrative work opportunity, more shared leisure outside the home and later the material removal of gaming access through selling his gaming computer. At the same time, we could have equally positioned Pavol's case as healing comorbidity, given the role of alcohol-related problems, or as an axiological shift, given the increased value of work, relational stability and non-gaming leisure. In other words, Pavol's natural recovery can be interpreted through at least three main paths (axiological shift, healing comorbidity and environmental transformation), which involve separate internal multicausal mechanisms.

Likewise, in the case of Gunho, parallel recovery paths were synergetically involved. Although ADHD diagnosis and medication served a central role in the process, the recently started master's programme—for which he expresses great passion—appears to have an impact as well, thus presenting similar axiological and environmental transformations to those of Pavol's. Unlike Pavol, however, Gunho had also received general psychological counselling for approximately 1 year, which may have contributed to his gradually evolving self-regulation skills (more about this below).

Regarding the reflexive transformation path, we see—and expect for future cases—a pattern where it is strongly connected to gradual regulation: reflexive transformation appears to manifest at least as a partial outcome of some gradually improving regulation. Like Gunho, Lauri had participated in expert counselling and even peer support groups, which he nonetheless found unhelpful and not sufficient for recovery. However, efforts like treatment involvement, even when unsuccessful, can contribute to (and mirror) progressing awareness and improvements of self-regulation—the transformative moment, as in Lauri's case, serves a critical function but is likely related to past developments that may be beyond the perceptual scope, even for Lauri himself.

Based on the present data, natural recovery from gaming-related health problems involves multiple collectively contributing factors. Within the scope of the present research design, no participants had received successful treatments explicitly targeting gaming behaviour. However, some cases did involve therapeutic interventions. These were typically not focused on gaming-related problems and instead addressed a broader spectrum of health and life challenges. Participants reported that these interventions were ineffective or insufficient in addressing their gaming but may still have had indirect effects—for example by contributing to gradual improvements in self-regulation skills or to axiological changes. Such potential influences cannot be observed within the present study design.

## 6. Discussion

This case series contributes to the understanding of how natural recovery unfolds in gaming-related problems by identifying six interrelated pathways: axiological shift, social enforcement, gradual regulation, reflexive transformation, healing comorbidity and environmental transformation. Across the cases, recovery was less characterized by abrupt resolution than by incremental adjustments in daily structures, social roles, self-regulation, comorbid difficulties and value hierarchies. Natural recovery in GD may be closely tied to shifting life structures rather than solely to changes in gaming behaviour itself.

These findings align with the broader natural recovery literature, which has described recovery as a gradual and multidimensional process shaped by shifts in environmental conditions or life context, rather than by individual willpower or self-control [22,23,29]. Prior research has indicated that the resolution of problem behaviours may be associated with life-course role transitions, processes of ‘maturing out’ [26] and increasing responsibilities, and that multiple pathways may lead to similar recovery outcomes [22,36]. Lauri's trajectory followed a similar pattern, as interesting academic commitments restructured his time, followed by ignited passion for exercise and new social engagements. Gaming did not disappear—it moved. Recovery emerged from the realignment of relational structures, role expectations and resource conditions [38] rather than from effort directed at gaming alone.

Moreover, the natural recovery literature has shown that addictive trajectories do not necessarily follow a chronic and progressive course; a substantial proportion may present as temporally limited, single-episode phenomena [23,29,36]. The present cases reflect a comparable pattern. Kangmin's gaming problems were largely confined to his graduate school years; Gunho's most intensive period coincided with the sudden absence of structure following university entrance; Gabriella's difficulties emerged specifically in the context of post-retirement time abundance. In each instance, the period of gaming problems was not a stable, enduring condition but a situationally contingent phenomenon that receded as the conditions sustaining it changed. At the same time, this very contingency implies potential vulnerability to recurrence: when the precipitating conditions re-emerge, so may the problematic behaviour. Pavol's case illustrates this well—his gaming problems resurfaced during the acute stress of his divorce proceedings, only to recede again once he removed access to the gaming platform. This pattern suggests that GD may, in some cases, unfold in a highly episodic and context-sensitive manner, consistent with recent research documenting temporal instability and periods of remission and recurrence [47,49].

Relatedly, the concept of recovery capital [37,42] provides a useful lens. For Beáta and Lauri, educational and vocational opportunities and structures served as key enabling conditions; for Kangmin, institutional expectations and social networks; for Pavol, financial stability and the restoration of his partnership. Gunho's case shows that improvements in executive functioning through ADHD treatment led to enhanced self-regulation. Gabriella, even in the absence of external environmental changes, gradually reduced gaming by repeatedly reflecting on and reassessing her preoccupation with top scores. The latter two cases illustrate how recovery capital can operate not only through material or relational resources but also through cognitive and emotional capacities, consistent with White & Cloud's [42] concept of human recovery capital: problem-solving capacity, self-efficacy and a sense of meaning and purpose in life.

Beyond structural and resource-based factors, GD recovery also raises questions about what constitutes a successful outcome. Natural recovery research has long acknowledged that recovery does not necessarily require complete abstinence; moderate use has often been recognized as a legitimate recovery outcome [20,32,57]. In substance-related research, moderate use typically refers to reduction to non-hazardous levels, defined in terms of quantity and frequency [58]. In the cases examined in the present study, changes in gaming were not simply about time spent or frequency but—perhaps more critically—involved shifts in meaning, purpose and structural repositioning in everyday life. This contributes tentative answers to our underlying research question by suggesting that meanings and values attached to gaming not only reflect a passion–pathology axis but can also serve as more general identity- and life-changing forces.

In our cases, gaming-related problems were largely triggered by specific environmental configurations: particular game platforms, competitive social cultures and situational stressors that intensified regulatory or escapist functions. Recovery therefore involved a transformation in the relationship between gaming and life more generally—a dynamic previously discussed as ‘psycholudic development’ [59] and sociologically frameable into the ‘digital gaming relationship’ [60,61]. Gaming moved from being a central, life-organizing activity to a bounded practice embedded within a broader ecology of responsibilities and roles.

For some participants, gaming was not merely a leisure activity but a central element of identity linked to academic study and professional aspirations (e.g. [53,62]). When Gunho attempted complete abstinence, he experienced feelings of emptiness and identity confusion, which ultimately led to a moderated re-engagement with gaming. For Beáta, gaming was intertwined with creative and professional identity and recontextualized within her recovery trajectory. These cases are consistent with the established idea that complete disconnection from gaming may not always constitute the most appropriate recovery goal [63]. Gaming activities appear to shift along with diverse life changes, potentially serving both adaptive and maladaptive functions.

### 6.1. Limitations

The interpretation of these findings is subject to several constraints. The cases were grounded in participants’ self-identified problems; although participants reported substantial distress and were embedded in a clinician-involved longitudinal design, recovery trajectories were reconstructed mainly from interview materials where participants retrospectively narrated their gaming-related problems and perceived problem resolution. Interpretations of the nature of these problems and their resolution vary even among experts; for instance, our expert panel of three clinicians did not agree whether Lauri met GD criteria or not (during the first year). What matters more than disorder agreement, however, is that Lauri experienced unquestionably significant problems related to gaming, and his story reflects recovery pathways that resonate with our other (diagnosable and non-diagnosable) cases too.

Moreover, given our design, the study prioritizes depth of understanding over representativeness: the six recovery pathways should be interpreted as exploratory patterns derived from recurring configurations in the data rather than as a definitive classification system or evidence of prevalence. While cases were drawn from three countries and cultural contexts were reflected on, the limited number of participants per context constrains cultural analyses.

Because the broader longitudinal study remains ongoing, the present case series should be read as an interim programmatic analysis rather than a final account. Reporting this trajectory now allows an underdeveloped recovery phenomenon to be examined while remaining open to revision as additional longitudinal data become available. The main risk of such mid-study analysis is premature closure; accordingly, the pathways are presented as provisional interpretive patterns, not as exhaustive categories.

Another limitation is that the present case series does not systematically examine how game genre, structure or design affordances may shape recovery pathways. Differences between solo and multiplayer games, competitive and non-competitive play, or specific design features, such as rankings, progression systems and session length, may influence how gaming becomes problematic and how recovery unfolds [64–66]. Future work should examine more directly how these game-specific conditions interact with personal, relational and environmental factors in shaping transitions between problematic and non-problematic gaming.

Some participants engaged in psychotherapy or treatment for comorbid conditions that did not explicitly target gaming behaviour. Although such interventions were not designed to address gaming-related problems directly, their potential indirect contribution to recovery cannot and should not be excluded. The study was not designed to establish causal effects, and pathway classification reflects analytic interpretation rather than quantified estimation, leaving the indirect role of such therapeutic contact open for further investigation.

Finally, as the broader longitudinal project remains ongoing, the long-term stability of the reported recovery trajectories would benefit from further follow-up. Such research may further articulate these recovery pathways within a broader structural (e.g. [67]) or ecological (e.g. [68]) framework, examining how different levels of conditions interact and reconfigure over time.

## 7. Conclusions

This case series focused on natural recovery from gaming-related health problems. In the cases examined, recovery did not appear to unfold through a single dominant mechanism, but rather through overlapping configurations of personal, relational and contextual change across six pathways. It was not limited to direct modifications of gaming behaviour but closely tied to shifts in life structure, role expectations and value hierarchies. Gaming often did not disappear entirely but remained in a repositioned and more clearly bounded form within a broader life ecology.

Rather than suggesting a linear trajectory or uniform outcome, the findings indicate that recovery may be better conceptualized as a multilayered process shaped by differing conditions and relational arrangements: the quality and quantity of gaming may adapt to various life events and health situations. In this sense, the present study offers a conceptual stepping stone towards more comprehensive frameworks for understanding and supporting recovery in gaming-related health problems, including cases of formal disorder diagnosis. Whether such changes remain stable over time, and under what conditions they may shift or reverse, remains an open question.

## Supplementary Material



## Data Availability

Data are currently being deposited in the Finnish Social Data Archive and made available for scientific reuse in pseudonymous form upon project completion: https://services.fsd.tuni.fi/catalogue/series/106?tab=description&lang=en. Supplementary material is available online [69].

## Footnote

^1^
At Stage 1, the latter group was referred to as ‘amateur esports players’, but we use the term ‘competitive players’ to improve clarity.
