# Supplementary material for: Natural recovery and gaming disorder: a case series
Source: R Soc Open Sci. 2026 Oct 21;13(10):262017. doi: 10.1098/rsos.262017 (PMC13682888; doi:10.1098/rsos.262017)
Supplement: Supplementary file 1 [file rsos.262017.zip › rsos.262017/rsos262017_si_001.pdf]

Appendix 1. Stage 1 Methods

The present case series draws on the methodological framework of the Stage 1 registered report, *Reconstructing Gaming Disorder: A Taxonomy by Registered Report*. The Stage 1 protocol was designed as a longitudinal multi-site study of intensive gaming in Finland, Slovakia, and South Korea. A full overview of the registered methodological workflow—including recruitment groups, data streams, timing, and analytic workflow—is provided in Figure 1 of the Stage 1 registered report (Dienes, 2023).

The Stage 1 protocol recruited participants through purposive homogeneous sampling into three groups of specific analytic interest: treatment-seekers, amateur esports players, and gaming adolescents. The present case series draws only on the adult participant groups: treatment-seekers and amateur esports players. In the registered protocol, treatment-seekers were defined as participants who had sought help for gaming from professionals such as healthcare providers, psychiatric or psychological services, or therapy centres, with gaming as a primary motive for treatment-seeking. Amateur esports players were defined as participants who self-identified as players of esports games and played 30 hours per week or more, while reporting no self-perceived gaming-related health problems. Recruitment goals and achieved sample sizes by country and group are presented in Table A1.

Table A1. Recruitment Goals and Achieved Sample by Country and Group

	Finland*	Slovakia*	South Korea	Total
Treatment-seekers (goal: n=45-60)	n=18	n=15	n=19	n=52
Amateur esports players (goal: n=15-30)	n=5	n=14	n=7	n=26

Note. The sampling frame for treatment-seekers was extended to include neighboring Nordic (for Finland) and Central European (for Slovakia) countries after completing local recruitment (nFinland=16; nSlovakia=5).

Across the broader Stage 1 project, participants are followed for 36 months. Adult participants receive annual clinical and phenomenological interviews.

Clinical interviews were conducted every 12 months by a licensed psychiatrist or clinical psychologist in each country and are designed to contextualize gaming within the participant's broader mental health situation. Phenomenological interviews are conducted annually by one or two team members using the Phenomenology of Play interview frame (Supplement 4 of the Stage 1 report). Participants also provide diary-like entries every four months (nine entries over three years), including open narrative content, gaming logs, and health-related scales.

Data were first analyzed following the principles of interpretive phenomenological analysis (IPA; Smith et al., 2022), a qualitative approach that prioritizes idiographic scrutiny—detailed, case-by-case analysis of how individuals make sense of their lived experience—of each participant. For each transcript, coding was carried out by one interviewing researcher and one non-interviewing researcher; a third researcher also read the transcript, and the three researchers met to discuss and resolve discrepancies. When agreement was not reached, alternative interpretations were retained

and reported. For follow-up interview transcripts, the analysis focused on longitudinal thematic changes, including narrative changes, participant and researcher reinterpretations, and the identification of stability over time (McCoy, 2017).

The present article does not reproduce the full Stage 1 methodology. The specific screening, eligibility criteria, pathway coding, and case selection procedures used for the present case series are reported in the main Data and Methods section.

Appendix 2. Coding and Pathway Derivation

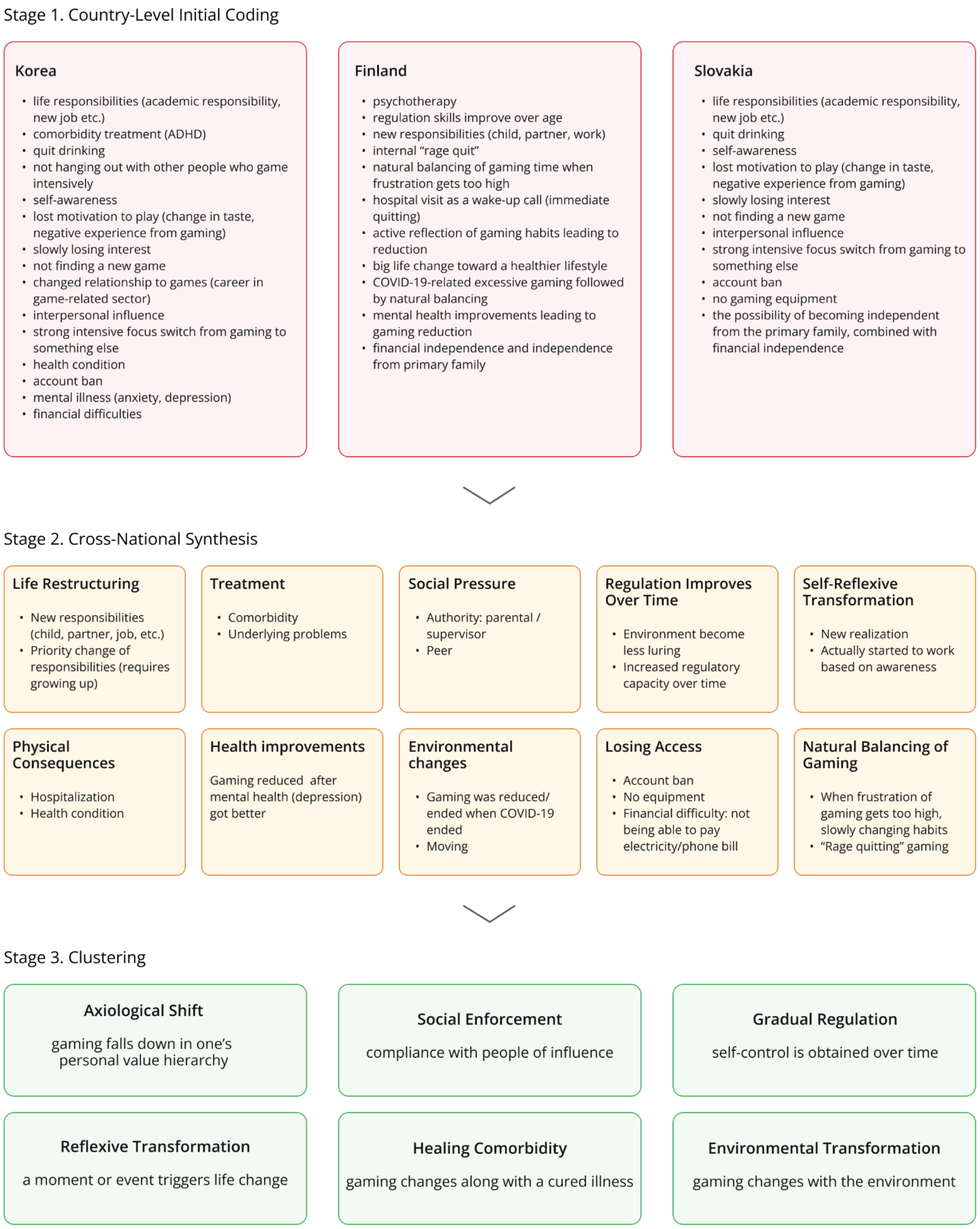

Figure A1. Analytical Process from Initial Coding to Recovery Pathways.

This figure illustrates the three-stage analytic procedure: country-level initial coding, cross-

national mechanism synthesis, and analytical clustering into six non-mutually exclusive recovery pathways.

Appendix 3. Treatment Histories and Gaming-Specific Treatment Status Across Cases

Table A2. Treatment Histories and Gaming-Specific Treatment Status

	Group	General Treatment History	Formal or Sufficient Gaming-Specific Treatment
Beáta	Esports	No, OCD diagnosis and self-reported aggression/meltdowns, but does not state whether she received counseling, psychiatric care, medication, or other treatment.	No. Never considered.
Kangmin	TS	Yes. Kangmin received self-analytic counseling approximately 5–7 years ago, for fewer than 50 sessions over about two years. This was not sought for gaming-related problems, but for broader self-understanding and relational difficulties with a romantic partner at the time. He also visited a clinic for work-related stress and sleep difficulties and took prescribed medication for approximately six months.	No. He considered gaming-related help, including addiction-related services, counseling centers, or hospitals, but did not follow up.
Gabriella	TS	No, Gabriella wanted help to reduce gaming but felt too embarrassed to discuss the problem with a clinician. She participated in the study partly as an opportunity to consult the clinicians in the study. She had a consultation meeting with a clinician at the start of the study.	No. She looked for help but did not receive formal or sufficient gaming-specific treatment. However, over the study years, she says that participating and regularly talking to clinicians has helped her reflect on her gaming and

			better control it now.
Lauri	TS	Yes. Lauri sought help from gaming disorder support groups after identifying gaming as a problem. Earlier he suffered from depression and gained expert clinical support for that, which also overlapped with him having identified gaming as a problem and the need to discuss it. Prescribed medication targeted depression, nonetheless.	No, not sufficient. Although he sought gaming-related help, he did not find these supports resolving the problem. His recovery is primarily attributed to a later reflexive transformation following repeated school-related failure.
Gunho	TS	Yes. Gunho sought counseling after experiencing gaming-related distress.He received university counseling and later external counseling, which focused broadly on self-understanding, achievement difficulties, interpersonal patterns, and developmental history. He was later diagnosed with ADHD and began ADHD medication.	No, not as formal or sufficient gaming-specific treatment. Although his help-seeking was prompted partly by gaming-related distress, the counseling he received was general psychological counseling rather than a structured intervention specifically targeting gaming behavior.
Pavol	TS	Yes. Pavol reported attending psychotherapy regularly during the study period.	No, no formal or sufficient gaming-specific treatment. Although gaming was mentioned as one topic in therapy, the primary focus concerned broader life difficulties.
